# A Six-Year Study on Epidemiology of Electrical Burns in Northern Iran: Is It Time to Pay Attention?

**DOI:** 10.29252/wjps.8.3.365

**Published:** 2019-09

**Authors:** Mohammad Tolouie, Ramyar Farzan

**Affiliations:** Guilan Road Trauma Research Center, Guilan University of Medical Sciences, Rasht, Iran

**Keywords:** Epidemiology, Electrical burn, Iran

## Abstract

**BACKGROUND:**

Although electrical burns are less prevalent than other types, they put socioeconomic burden on communities, yielding higher mortalities. Therefore, the frequency and causes of electrical burns in the largest burn center in northern Iran were studied.

**METHODS:**

All patients with electrical burn injuries admitted to Velayat Hospital, Rasht, Iran participated in this descriptive cross-sectional study. The data collection tool was a checklist including demographic data, damage mechanism, voltage classification (high or low), injured organ, ICU need, length of stay (LOC), electrical burn severity (degree and area of burns based on TBSA), surgical interventions, and return to work. All data were gathered through HIS system and analyzed.

**RESULTS:**

Most electrical burns occurred in men (99.4%) and most of whom had electricity-related jobs (26%). The majority of victims had third-degree burns (63%), and electrical current-induced burns in entry points occurred in the upper and lower extremities, head and other organs ranked the first to fourth, respectively. Most burns happened due to abrupt contact with electrical current (83.33%) in routine home activities (52.78%). The mean LOC was 8.73 days, suggesting that LOC increased significantly, if the electrical current entered the body through lower extremities, while it decreased significantly, if the electrical current exited through lower extremities.

**CONCLUSION:**

The majority of electrical burn victims were men. Most burns occurred in urban communities in summer. Most people were affected by high voltage electricity.

## INTRODUCTION

Electrical injuries are among the most important health problems worldwide. Despite the numerous efforts to reduce the number of electrical damages, their incidence has an increasing trend.^1^^,^^2^ Electrical burns account for about 3-6% of hospitalization in burn wards.^3^ In the United States, electrical burns are responsible for 4-6.5% of hospitalization in burn wards, which annually cause 1,000 deaths.^4^ The incidence of electricity-induced injuries is higher in rural areas and outskirts in developed countries compared to developing nations.^5^

Electrical damages in our country is much more frequent than developed countries and has many negative socioeconomic impacts.^6^ Although electrical burns are less common than other types of burn, they are among harmful damages and cause a higher mortality rate.^7^ They are the third leading cause of burns, following scald and flame-induced burns that put heavier socioeconomic burdens on communities in spite of advanced therapeutic modalities and decreasing mortality rates than other types of burns.^8^ The victims of electrical burns are often small children, adolescents and young people in working age.^2^^,^^9^^,^^10^

Electrical burns often cause damage to both parts of skin (superficial, deep) and also deeper tissues, and can lead to large areas of necrosis.^11^ The mechanism of electrical burn damage is complex and unknown, and depends on several factors such as damaged area, contact time, and electrical current intensity.^2^ These burns are divided into four groups according to the damage mechanism including (i) direct contact with an electric heater, (ii) indirect contact with a flame, (iii) indirect contact with electricity, and (iv) indirect contact with an electric-thermal equipment.^12^

These damage are also divided into three categories in terms of the mechanism including (1) electrical damage; (2) damage to the flexor surfaces of the body, such as the armpit; and (3) heat damage caused by a flame, the damage caused by the combustion of clothing. The severity of tissue damage can be classified according to the electrical intensity (low and high voltage), the type of current (direct or indirect), the affected part of the body, the contact time, and simultaneous secondary damages.^12^ They can also be classified into two main groups, burns with a current above 1000 volts and below 1000 volts.^3^

Low voltage-induced burns result in inability and destruction in the affected site tissue, while high voltage-induced burns cause systemic damages.^13^ Electricity-caused damages have immediate consequences in short- term and require extensive medical intervention, and their complications are much deeper and more difficult to detect in the long run.^14^ Since the exact diagnosis of electrical burn severity is difficult, and many people underestimate it,^15^ this study aimed to investigate electrical burns and associated factors in burn patients at Velayat Burn Center of Guilan University of Medical Sciences, Rasht, Guilan, Iran.

## MATERIALS AND METHODS

This descriptive cross-sectional study enrolled 108 patients, suffering from electrical burn injuries admitted to Velayat Teaching Hospital in Rasht (Burn Center of Guilan Province, Northern Iran) in a six year-period (2011-2016). The data collection tool was a checklist containing the demographic data (age, sex, level of education, location of injury), damage mechanism, voltage classification (high or low), intensive care unit (ICU) need, length of stay, electrical burn severity (degree and area of burns based on Total Body Surface Area: TBSA), surgical interventions (fasciotomy, escharotomy, amputation), and return to work. Patients with partial information, incomplete treatment period, and discharged against medical advice were excluded from the study.

Once the patient was admitted to the hospital, the degree of burn and affected surface area of the body (TBSA) were determined, and then tests of cardiac rhythm and kidney function (intake/output, myoglobinuria, blood urea nitrogen (BUN) and serum creatinine levels) and if needed, resuscitation were conducted. In case of thermal damage, Parkland’s formula was used. Adequate resuscitation was evaluated by monitoring the amount of urine output. In the first line, oral or injectable non-steroidal anti-inflammatory drugs were used routinely to manage the pain of the patients, and in the second line, opiate drugs were taken. 

Daily dressing, debridement, and skin grafting were done as needed. Patients were carefully evaluated for compartment symptoms and, if necessary, fasciotomy or delayed guillotine amputation was performed and, if necessary, the amputation site was corrected. Amputations were performed only in sterile conditions, and antibiotic therapy was undertaken for those who had positive culture samples. All data were collected through HIS system and analyzed using SPSS software (version 21, Chicago, IL, USA) and descriptive statistics tests.

## RESULTS

The whole 108 patients including 102 male patients (94.4%) and 6 female patients (5.6%), aged between 1 and 72 years with a mean age of 31.56 years, participated in the study. The most common frequency of burns was found in patients with diploma and higher educational level, followed by illiterate patients and those with elementary education. Most people with burns had electricity-related jobs (26%). The burns occurred mostly in urban communities in summer months. While the third-degree burns took place more frequently (63%), the surface area of burn was commonly low (0-19%, n=97). 

Burns induced by electrical current in entry points were mainly observed in upper and lower extremities, head and other organs ranked the first to fourth, respectively, while electrical burns in exit points occurred inversely except for the head. Most victims were damaged with high voltage electricity (n=68, 62.96%). The burns occurred predominantly due to abrupt contact with electrical current (n=90, 83.33%) in daily routine activities at home (52.78%, Table 1). 

**Table 1:** Frequency of the study variables in patients with electrical burns admitted to Velayat Hospital, Rasht, Iran

**Variable**		**Frequency**	**Percent**
Education	Illiterate	24	22.22
	Elementary	4	3.70
	Diploma and higher	80	74.07
Season	Spring	34	31.48
	Summer	39	36.11
	Autumn	25	23.15
	Winter	10	9.26
Accident location	Urban areas	80	74.07
	Rural areas	28	25.93
Burn surface area	0-19	97	89.8
	20-39	7	6.5
	40-59	3	2.8
	60-100	1	0.9
Burn degree	First	2	1.9
	Second	28	25.9
	Third	68	63
	Fourth	10	9.3
Electrical current Entry point	Upper extremities	96	88.89
	Lower extremities	9	8.33
	Head	2	1.85
	Others	1	0.93
Electrical current exit point	Upper extremities	21	19.44
	Lower extremities	29	26.85
	Head	2	1.85
	Others	56	51.85
Accident cause	Abrupt contact with electricity current	90	83.33
	Accidents at work	9	8.33
	Lightning	9	8.33
Accident place	Home	57	52.78
	Workplace	19	17.59
	Shop	17	15.74
	Others	15	13.89

The length of stay varied from 1 to 45 days, with the mean of 8.73 days. Poisson regression analysis showed that the length of stay in men was significantly less than that in women. Age had an inverse relationship with the length of stay, so that the probability of hospitalization decreased significantly (0.007) with the one year increase in age. Also, people with electrical burns above 50% and 75% showed a 2.5 and 5.3 time increase, respectively, in the probability of length of stay, which was statistically significant. 

There was no significant relationship between the second- and third-degree burns with length of stay, while length of stay in the patients with the fourth-degree burns was 6.1 times higher than that in others. If the electrical current entered the body through lower extremities, the length of stay increased significantly, while the length of stay declined significantly, if the electrical current exited the body through lower extremities. 

## DISCUSSION

According to the World Health Organization (WHO), 195,000 people die annually because of burns. Most deaths occur in low- and middle-income countries and half of them in the Southeast Asia.^16^ Among several types of burns, electrical burn is one of the most disturbing kinds of trauma.^17^ Although patients with electrical burn injuries account for less than 5% of all admissions in the burn centers globally, they bring out significant morbidities and mortalities.^8^^,^^18^ Similar to many previous researches, most patients employed in the current study are young men in working age. 

This is likely to be the result of recruiting men in occupations that are related to electrical currents and heavy equipment and machinery. Furthermore, burn accidents may happen due to inadequate equipment and training.^19^^-^^21^ Human errors can also be considered to be among the negative influencing factors, but appropriate training can eliminate their effects, as demonstrated in various studies.^22^^,^^23^ Our study showed that the electrical current entered the body more commonly through upper and lower extremities, and head ranked the first to third, respectively. 

Li and colleagues found that head, face, and neck were the most frequent location of electrical burns.^24^ In the present study, most patients were injured with high-voltage electrical current (n=68, 62.96%), while Li *et al.* showed that most of the patients were affected by low voltage.^24^ The present study found that the most common burns happened through a sudden contact with the electrical current. It may be because of the lower level of safety standards in that environment in developing countries than developed nations. 

Other studies have confirmed that electrical accidents take place more commonly in the indoor settings in the countries such as Brazil or Nigeria compared to European countries. These regional differences suggest that prevention safety standards have not been established in all parts of the world, and much work has still to be done.^9^^,^^25^ In electricity-damaged areas (electricity entry and exit points), third- and fourth-degree burns with full skin necrosis are seen, and sometimes with tissue necrosis under the skin such as fascia, nerves, muscles, tendons, vessels and bones.

Normally, the area involved in these parts is very small, and does not include more than 1% of the body surface. However, deep muscles in most cases and even superficial muscles in some extent are burned due to severe heat. The heat generated directly is related to the resistance of that part of the body and the voltage. In high voltage injuries, much more serious injuries and burns happen in muscles.^26^^,^^27^ According to results of the study, high voltage electrical injuries were among the most common reasons for admitting patients. 

Considering that most of our study patients had burn under 19% based on TBSA, and most cases had the third-degree burns, higher length of stay could be justified. In Brazil, a similar study showed a relationship between high-voltage burns with severity of injuries, clinical complications, and amputations. Moreover, the length of stay was higher in subjects with amputation and compartment syndrome.^8^^,^^28^ In our study, less than 1/4 of patients needed fasciotomy and admission to the intensive care unit. These results are consistent with the findings of other studies.^29^^,^^30^

This could be attributed to the timely referral of patients at the very early minutes after burn to the hospital and their proper management. In our study, almost all patients were able to return to their job after the recovery process. This can be attributed to monitoring and early advanced resuscitation, more improved management of wound and surgery, and early grafting. Contrary to our findings, Noble *et al.* stated that patients had remarkable problems when returning to their work. Only 23% of them were able to return to their job and perform the same tasks, while 32% of them could not return to work at all. The rest of the patients were forced to work in the same job by modifying their duties or in another occupation.^31^

The current study also found that most electrical accidents occurred in urban areas. This contradicts the findings of the study by Rao *et al.*, who introduced rural environments more insecure than urban settings because of not observing safety precautions.^32^ The more frequent electrical accidents in summer months compared to other seasons may be due to sweating increases in summer, which in turn reduces skin resistance and thus raises the flow of electrical current through the body.^33^ Indeed, electrical damages happen commonly in urban regions and hot weather during the theft of expensive copper cables,^2^^,^^34^ or during illegal cabling in order to not paying tariffs assigned to use cooling devices such as air conditioners in developing countries with many socioeconomic problems. 

Poisson regression analysis showed that the length of stay in men was significantly less than that in women due to that men have lower degree of burn because of their thicker skin leading to faster treatment. The age had an inverse relationship with the length of stay, so that the probability of hospital attendance decreased significantly (0.007) with one year increase in age. Also, people with electrical burns above 50% and 75% raised the probability of their length of stay as 2.5 and 5.3 times, respectively, which was statistically significant. 

A study conducted by Lunawat *et al.* showed that the length of stay was between 4 and 83 days with mean length of 23.53 days. The patients with high TBSA had longer length of stay.^35^ Our study found that the length of stay in the patients with the fourth-degree burns was 6.1 times higher than that in others. Ghavami *et al.* in their study showed that the length of stay was higher at high voltages.^2^ Our study found that the majority of electrical burn victims were men. Most burns occurred in urban communities in summer months. 

The people suffering from electrical burn were predominantly damaged by high voltage electricity, so burn prevention should be the top priority, and government programs should focus on the safety and proper management of electrical appliances. Occupational safety acts must be revised, and employers must respect these regulations. Workers exposed to electrical equipment must be fully trained and certified.

## CONFLICT OF INTEREST

The authors declare no conflict of interest.
